# Clinical proteomic analysis of scrub typhus infection

**DOI:** 10.1186/s12014-018-9181-5

**Published:** 2018-02-14

**Authors:** Edmond Changkyun Park, Sang-Yeop Lee, Sung Ho Yun, Chi-Won Choi, Hayoung Lee, Hyun Seok Song, Sangmi Jun, Gun-Hwa Kim, Chang-Seop Lee, Seung Il Kim

**Affiliations:** 1 Drug & Disease Target Team, Korea Basic Science Institute (KBSI), Cheongju, 28119 Republic of Korea; 2 Center for Convergent Research of Emerging Virus Infection, Korea Research Institute of Chemical Technology (KRICT), Daejeon, 34114 Republic of Korea; 3 Department of Bio-Analytical Science, University of Science and Technology (UST), Daejeon, 34113 Republic of Korea; 4 Division of Life Science, Tunneling Nanotube Research Center, Korea University, Seoul, 02841 Republic of Korea; 5 Department of Internal Medicine, Chonbuk National University Medical School, Jeonju, 54986 Republic of Korea; 6 Biomedical Research Institute of Chonbuk National University Hospital, Jeonju, 54907 Republic of Korea

**Keywords:** Scrub typhus, Clinical proteomics, *Orientia tsutsugamushi*

## Abstract

**Background:**

Scrub typhus is an acute and febrile infectious disease caused by the Gram-negative α-proteobacterium *Orientia tsutsugamushi* from the family Rickettsiaceae that is widely distributed in Northern, Southern and Eastern Asia. In the present study, we analysed the serum proteome of scrub typhus patients to investigate specific clinical protein patterns in an attempt to explain pathophysiology and discover potential biomarkers of infection.

**Methods:**

Serum samples were collected from three patients (before and after treatment with antibiotics) and three healthy subjects. One-dimensional sodium dodecyl sulphate–polyacrylamide gel electrophoresis followed by liquid chromatography-tandem mass spectrometry was performed to identify differentially abundant proteins using quantitative proteomic approaches. Bioinformatic analysis was then performed using Ingenuity Pathway Analysis.

**Results:**

Proteomic analysis identified 236 serum proteins, of which 32 were differentially expressed in normal subjects, naive scrub typhus patients and patients treated with antibiotics. Comparative bioinformatic analysis of the identified proteins revealed up-regulation of proteins involved in immune responses, especially complement system, following infection with *O. tsutsugamushi*, and normal expression was largely rescued by antibiotic treatment.

**Conclusions:**

This is the first proteomic study of clinical serum samples from scrub typhus patients. Proteomic analysis identified changes in protein expression upon infection with *O. tsutsugamushi* and following antibiotic treatment. Our results provide valuable information for further investigation of scrub typhus therapy and diagnosis.

**Electronic supplementary material:**

The online version of this article (10.1186/s12014-018-9181-5) contains supplementary material, which is available to authorized users.

## Background

Scrub typhus (tsutsugamushi disease) is a mite-borne infectious disease caused by the Gram-negative α-proteobacterium *Orientia tsutsugamushi* that is transmitted through the bite of an infected chigger (Trombiculidae mite) [1]. After a bite from an infected chigger, a characteristic necrotic inoculation lesion, termed eschar, can develop, and the microorganism then spreads through the lymphatic fluid and blood, causing systemic manifestations including fever, headache, myalgia, lymphadenopathy and skin rash. Especially in untreated cases, patients develop complications with systemic involvement and disseminated vasculitis, including septic shock, acute respiratory distress syndrome, acute renal failure, meningitis, myocarditis, gastrointestinal bleeding and multiorgan dysfunctions [2, 3]. Scrub typhus is endemic in the ‘tsutsugamsuhi triangle’ area, which extends from Northern Japan and far Eastern Russia in the North, to Northern Australia in the South, and to Pakistan and Afghanistan in the East [4–6]. Over one billion people are currently living in risk areas, and approximately one million cases occur annually worldwide [7]. The average case-fatality rate is usually ~ 10%, but can be as high as 35% if antimicrobial treatment is delayed. In the pre-antibiotic era, case-fatality ratios may have been as high as 50% [8]. Therefore, early diagnosis of scrub typhus is essential to prevent complications and to reduce the mortality rate.

The main goal of proteomic studies is to elucidate protein expression and identify changes under the influence of biological perturbations such as diseases or drug treatment [9, 10]. The information obtained from such analyses provides valuable information on pathophysiology and potential diagnostic biomarkers of disease [11]. Since the genome sequence of *O. tsutsugamushi* was published [12], several proteomic studies have focused on identifying proteins. The first proteomic analysis involved 2D liquid chromatography-tandem mass spectrometry (LC–MS/MS)-based comparison of protein expression, and 14 proteins were shown to be differentially expressed in antibiotic-sensitive and -insensitive *O. tsutsugamushi* [13]. Kishimoto and colleagues also performed shotgun proteomics analysis of *O. tsutsugamushi*, which revealed specific characteristics of this obligate intracellular bacterial species, and identified potential immunogenic factors such as type IV secretion system proteins [14]. More recently, comprehensive analysis of global gene and protein expression in *O. tsutsugamushi* in fibroblasts and macrophages showed how the pathogen responds in different types of host cells [15].

Although previous proteomic studies on *O. tsutsugamushi* provided some evidence about the pathophysiology of scrub typhus, information about diagnostic markers is lacking. Currently, complement fixation is one the earliest tests for diagnosis of scrub typhus [2, 16, 17]. At present, there is no diagnostic test of pathogenic antigen generated by *O. tsutsugamushi*. Moreover, proteomic analysis of *O. tsutsugamushi* directly isolated from scrub typhus patients has not been successfully performed, presumably because *O. tsutsugamushi* proteins are present at low concentrations in the blood. In the present study, as the first step in proteomic analysis of scrub typhus patients, we investigated the serum proteome to elucidate physiological changes caused by infection, both before and after antibiotic treatment.

## Methods

### Patients and clinical samples

The diagnosis of patients with scrub typhus was confirmed by a positive IgM titre ≥ 1:160 against *O. tsutsugamushi*, or a fourfold or greater rise in the indirect immunofluorescence assay (IFA, Green Cross Reference Lab., Yongin, South Korea). Detailed patient information is listed in Table 1. Blood was collected from scrub typhus patients, and peripheral blood mononuclear cells (PBMCs) were prepared for PCR validation of *O. tsutsugamushi* infection. For proteomic analysis, blood serum samples were prepared from three scrub typhus patients before and after antibiotic treatment. Doxycycline 200 mg/day was used to treat for 5 to 7 days. First blood sample was collected at emergency room before antibiotics administration and second sample was 5 to 7 days after antibiotics. Sera from three healthy subjects were used as negative infection controls.

**Table 1:** Clinical characteristics of patients with scrub typhus

	Patient 1	Patient 2	Patient 3
Age (years)/sex	80/F	58/M	70/F
Systemic symptoms	Fever, chills, headache, myalgia	Fever, chills, myalgia, nausea/vomiting	Fever, myalgia
Skin rash	+	+	+
Eschar	+	+	+
Laboratory findings			
WBC	6850	5940	4560
Hemoglobin	12.5	13.7	10.7
Platelet	159,000	114,000	106,000
AST	119	69	124
ALT	32	46	106
Total bilirubin	0.32	0.25	1.16
Albumin	3.8	3.3	3.8
PT (s)	11.2	10.2	11.2
PT (INR)	1.06	0.97	10.6
aPTT (s)	24.3	36.7	33.1

*WBC* white blood cell, *AST* aspartate aminotransferase, *ALT* alanine aminotransferase, *PT* prothrombin time, *INR* international normalized ratio, *aPTT* activated partial thromboplastin time

### PCR

Standard PCR was performed with 20 ng/µl DNA from PBMCs. For nested PCR, 2 µl of a 100-fold diluted standard PCR mixture was used as template. Primers used for PCR amplification of a gene encoding a 56 kDa protein were as follows: standard PCR (862 bp), 5′-CAATGTCTGCGTTGTCGTTGC-3′ (forward) and 5′-ACAGATGCACTATTAGGCAA-3′ (reverse); nested PCR (509 bp), 5′-CCAGGATTTAGAGCAGAG-3′ (forward) and 5′-CGCTAGGTTTATTATCAT-3′ (reverse).

### Preparation of clinical serum samples for proteomic analysis

For proteomic analysis, albumin and IgG were removed from clinical serum samples using ProteoPrep Immunoaffinity Albumin and IgG Depletion Kit (Sigma-Aldrich, St. Louis, MO, USA). After column equilibration, 100 µl of serum sample (diluted with 50 µl of dilution solution) was loaded on the column and incubated for 10 min at room temperature. The eluate was collected by centrifugation at 8000×*g* for 1 min, and reapplied to the same spin column. The second elute was collected and used for proteomic analysis.

### Sodium dodecyl sulphate–polyacrylamide gel electrophoresis (SDS-PAGE) and tryptic digestion

Prepared clinical serum samples (10 µg) were resuspended in sodium dodecyl sulphate–polyacrylamide gel electrophoresis (SDS-PAGE) sample buffer (1 M TRIS–HCl pH 6.8, 10% SDS, 1% bromophenol blue, glycerol, β-mercaptoethanol) and boiled for 10 min. Samples were separated by 12% SDS-PAGE. The gel was stained with Coomassie Brilliant Blue R-250 and fractionated according to molecular weight. Tryptic in-gel digestion was conducted according to a previous procedure [18]. Digested peptides were extracted with extraction solution consisting of 50 mM ammonium bicarbonate, 50% acetonitrile and 5% trifluoroacetic acid (TFA), and dried. For LC–MS/MS analysis, samples were dissolved in 0.5% TFA.

### Proteomic analysis by LC–MS/MS analysis

Tryptic peptide samples (5 µl) were separated using an Ultimate 3000 UPLC system (Dionex, Sunnyvale, CA, USA) connected to a Q Exactive Plus mass spectrometer (Thermo Scientific, Waltham, MA, USA) equipped with a nanoelectrospray ion source (Dionex). Peptides were eluted from the column and directed onto a 15 cm × 75 μm i.d. Acclaim PepMap RSLC C18 reversed-phase column (Thermo Scientific) at a flow rate of 300 nl/min. Peptides were eluted by a gradient of 0–65% acetonitrile in 0.1% formic acid for 180 min. All MS and MS/MS spectra obtained using the Q Exactive Plus Orbitrap mass spectrometer were acquired in the data-dependent top10 mode, with automatic switching between full scan MS and MS/MS acquisition. Survey full scan MS spectra (m/z 150-2,000) were acquired in the orbitrap at a resolution of 70,000 (m/z 200) after accumulation of ions to a 1 × 10^6^ target value based on predictive automatic gain control (AGC) from the previous full scan. MS/MS spectra were searched with MASCOT v2.4 (Matrix Science, Inc., Boston, MA, USA) using the UniProt human database for protein identification. MS/MS search parameters were set as follows: carbamidomethylation of cysteines, oxidation of methionines, two missed trypsin cleavages, mass tolerance for parent ion and fragment ion within 10 ppm, *p* value < 0.01 of the significant threshold. The exponentially modified protein abundance index (emPAI) was generated using MASCOT, and mol% was calculated according to emPAI values [19]. MS/MS analysis was performed at least three times for each sample.

### Statistical analysis and bioinformatics

Analysis was performed only on proteins that were detected more than two times from triplicate experiments for each sample. Differentially expressed proteins among the three groups were identified using Kruskal–Wallis tests. Non-parametric Mann–Whitney U tests were also performed to identify differentially expressed proteins within each group. Proteins were considered significantly differentially expressed when the *p*-value was less than 0.05 as calculated using R (http://www.r-project.org). All identified proteins were subjected to query canonical pathway analysis using the Ingenuity Pathway Analysis (IPA) tool (https://www.qiagenbioinformatics.com/products/ingenuity-pathway-analysis/).

## Results and discussion

### Evaluation of clinical samples

Sera from three scrub typhus patients were used for proteomic analysis. We collected blood from patients before and after antibiotic treatment. To confirm the quality of clinical blood samples from scrub typhus patients, PBMCs were isolated from blood, and the presence of pathogen was evaluated by nested PCR. The results showed that all three patients were positive for a specific scrub typhus gene encoding a 56 kDa protein, confirming infection by *O. tsutsugamushi* (Additional file 1: Fig. S1).

### Proteomic analysis

**Fig. 1:**
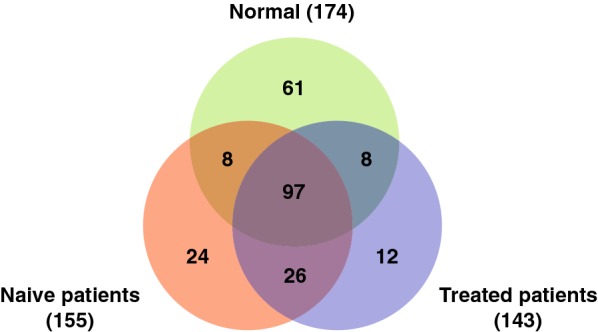
Identification of serum proteins in scrub typhus patients. The Venn diagram shows the number of proteins identified in the serum of normal subjects, naive scrub typhus patients and patients treated with the antibiotic doxycycline

To identify host proteins affected by scrub typhus infection, serum samples were prepared from normal subjects (controls), naive patients (before antibiotic treatment) and treated patients (after antibiotic treatment), then analysed by GeLC-MS/MS. From the proteomic analysis, 174, 155 and 143 human proteins were identified in the serum of normal subjects, naive scrub typhus patients and treated patients, respectively (Fig. 1 and Additional file 2: Table S1). Following infection with *O. tsutsugamushi*, expression of 70 proteins was significantly up-regulated, and expression of 94 proteins was down-regulated, compared with normal subjects (Table 2). After antibiotic treatment, 26 proteins were up-regulated and 36 proteins were down-regulated in treated patients relative to naive patients (Table 2). These proteins could be useful for investigating the pathophysiology of scrub typhus at the molecular level, and for discovering diagnostic biomarkers of *O. tsutsugamushi* infection. The quantitative results also revealed that the number of proteins expressed at similar levels in normal subjects and naive patients was 60 (26.8%), while the number between naive patients and treated patients was 113 (64.6%), indicating that protein expression was more similar between treated patients and naive patients than between treated patients and normal subjects (Table 2). This may suggest that treated patients are in the process of recovery, but not fully recovered.

**Table 2:** Summary of quantitative result of serum proteomic analysis

Fold change	No. of proteins
Normal subjects versus naive patients	
Naive patients only	50
Naive/normal > 2.0-fold	20
2.0-fold > naive/normal > 0.5-fold	60
Naive/normal < 0.5-fold	25
Normal subjects only	69
Total	224
Naive patients versus treated patients	
Treated patients only	20
Treated/Naive > 2.0-fold	6
2.0-fold > treated/naive > 0.5-fold	113
Treated/naive < 0.5-fold	4
Naive patients only	32
Total	175

### Comparative analysis of canonical pathways

Discovery of canonical pathways enriched in different conditions and investigation of their differences upon infection with *O. tsutsugamushi* could provide potential information on the pathophysiology of scrub typhus. To this end, the identified proteins were analysed using the IPA bioinformatics tool. Bioinformatic analysis revealed that the identified proteins are mainly involved in immune responses. In all groups, acute phage response signalling and complement system were ranked in the top five in the canonical pathway (Additional file 1: Fig. S2). In addition, proteins related to LXR/RXR activation, FXR/RXR activation and coagulation system were enriched in all samples (Additional file 1: Fig. S2).

**Fig. 2:**
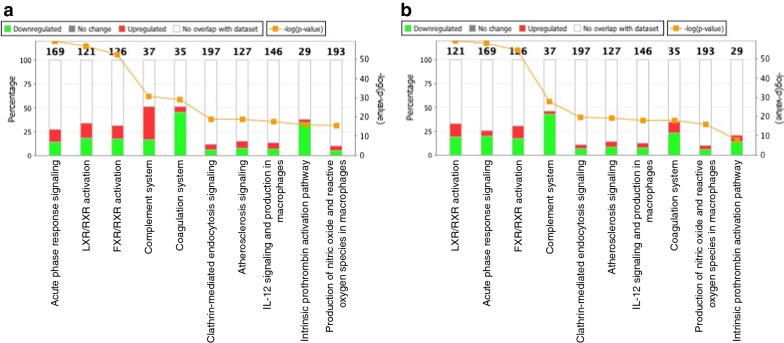
Top 10 canonical pathways most significantly altered in naive scrub typhus patients compared with normal subjects (**a**) and in patients treated with antibiotics compared with naive patients (**b**). The stacked bar chart displays the percentage of proteins up-regulated (red) and down-regulated (green), and of proteins not overlapping with the dataset (white), in each canonical pathway. The numerical value at the top of each bar represents the total number of genes in the canonical pathway. The secondary y-axis (right) shows the −log of the *p* value calculated by the Benjamini–Hochberg method

**Fig. 3:**
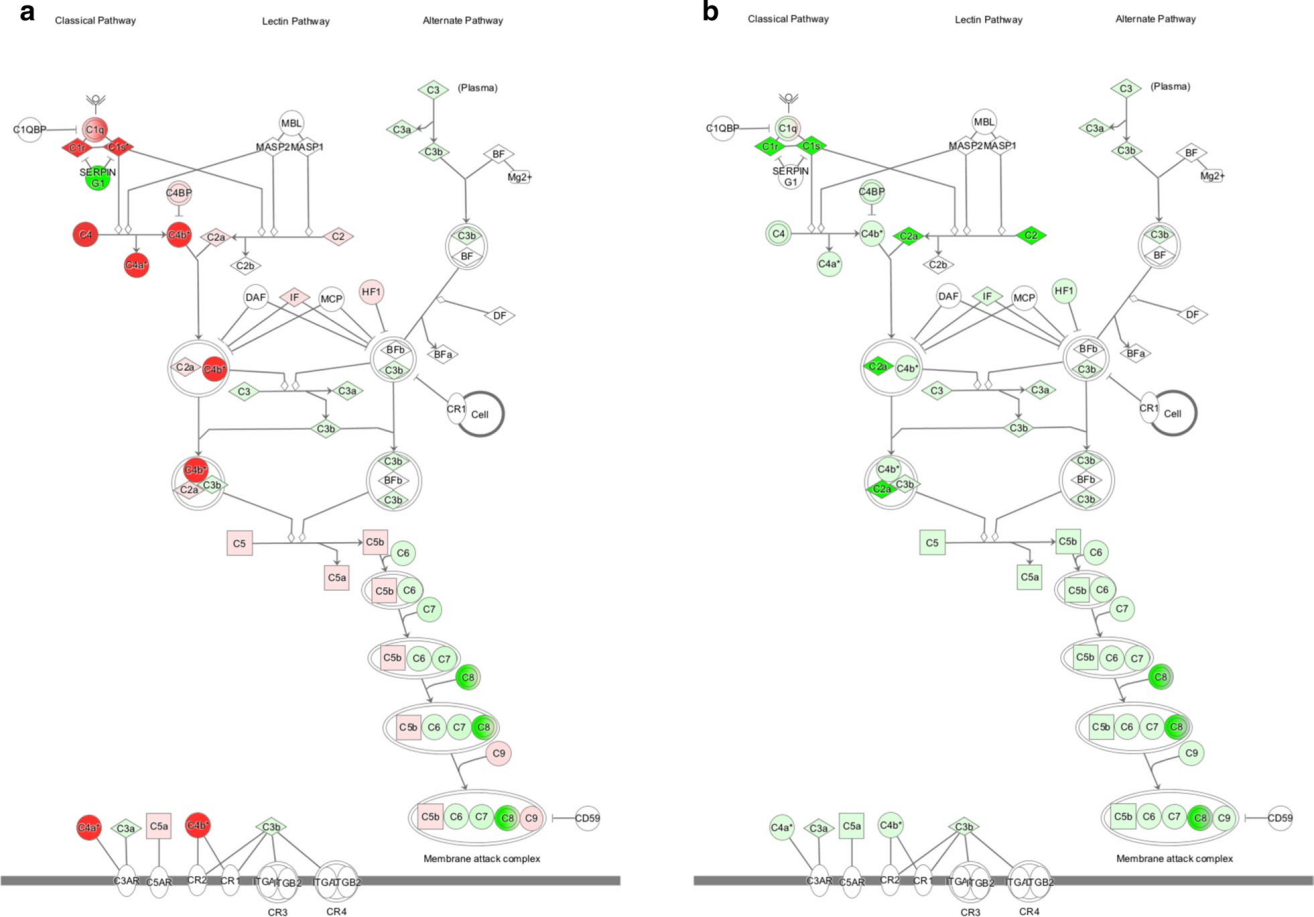
Complement system is a canonical pathway significantly altered in naive scrub typhus patients compared with normal subjects (**a**) and in patients treated with antibiotics compared with naive patients (**b**). Red indicates up-regulated proteins, and green indicates down-regulated proteins. The colour intensity corresponds to the degree of up- or down-regulation (fold change). White represents proteins known to be part of the pathway but not identified in the proteomic analysis

To better understand the differences in expression of proteins and changes in the canonical pathways following infection with *O. tsutsugamushi* and subsequent antibiotic treatment, we compared the identified proteins and their enriched canonical pathways. The results revealed dynamic changes in the expression of proteins involved in immune responses (Fig. 2). In particular, expression of proteins associated with complement system was up-regulated by infection with *O. tsutsugamushi* (Fig. 2a) and down-regulated by antibiotic treatment (Fig. 2b). Investigation of signalling pathways also confirmed that complement system was significantly activated in scrub typhus patients, and activity was down-regulated by antibiotic treatment (Fig. 3).

### Differentially expressed proteins

Next, we identified proteins displaying statistically significant differential expression between the three groups, and 32 differentially expressed proteins were identified (Table 3), of which 27 were up-regulated by infection with *O. tsutsugamushi* and down-regulated by antibiotic treatment. Representative proteins up-regulated in naive patients include serum amyloid proteins, complement component proteins, protein S100-A8 and C-reactive protein, which are mainly related to immune responses (Table 3). Up-regulation of immune responses is expected since scrub typhus infection induces a combination of non-specific symptoms that overlap with other infections [20]. Interestingly, platelet factor 4 was exclusively expressed in scrub typhus patients (Table 3). This small cytokine is secreted from platelets during platelet aggregation, and promotes blood coagulation [21, 22]. Recent studies reported that blood coagulation is activated by infection with *O. tsutsugamushi* [23–25]. Five proteins, serotransferrin, tetranectin, ficolin-3, selenoprotein P and adiponectin, were down-regulated in the serum of scrub typhus patients and up-regulated in patients treated with antibiotics (Table 3). Proteins displaying differential expression among normal subjects, naive patients and treated patients could be potential biomarkers for diagnosis and/or prediction of therapy responses during scrub typhus infection.

**Table 3:** List of differentially expressed proteins

Uniprot ID	Protein description	Protein mol %			GO Biological function^a^
		Normal subjects	Naive patients	Treated patients	
Up-regulated in naïve patients and down-regulated in treated patients					
P0DJI9	Serum amyloid A-2 protein	–^b^	0.8452	–	Acute-phase response
P0DJI8	Serum amyloid A-1 protein	–	0.6880	–	Acute-phase response
A0A0G2JPR0	Complement C4-A	–	0.6873	0.4188	Complement activation
P05109	Protein S100-A8	–	0.4644	–	Inflammatory response
Q5VY30	Retinol binding protein 4, plasma, isoform CRA_b	0.2306	0.4052	–	Glucose metabolism
P61769	Beta-2-microglobulin	–	0.3403	–	Antigen processing and presentation
P02776	Platelet factor 4	–	0.3367	–	Platelet activation
P07360	Complement component C8 gamma chain	0.1993	0.3285	–	Complement activation
P69891	Hemoglobin subunit gamma-1	–	0.3034	–	Blood coagulation
A0A0A0MSV6	Complement C1q subcomponent subunit B	0.0647	0.2777	0.1604	Complement activation
P02748	Complement component C9	0.1072	0.2378	0.1359	Complement activation
P02741	C-reactive protein	–	0.2212	–	Acute-phase response/Inflammatory response
P59665	Neutrophil defensin 1	–	0.1911	–	Innate immune response
O75636	Ficolin-3	–	0.1800	–	Complement activation
P01034	Cystatin-C	–	0.1682	–	Neutrophil degranulation/Defense response
Q06033	Inter-alpha-trypsin inhibitor heavy chain H3	–	0.1485	–	Platelet degranulation
P60709	Actin, cytoplasmic 1	–	0.1384	–	Platelet aggregation
A0A0G2JRQ6	Uncharacterized protein	–	0.0850	–	Unkown
A0A087X232	Complement C1 s subcomponent	–	0.0730	–	Complement activation
P00736	Complement C1r subcomponent	–	0.0461	–	Complement activation
Q9H2R5-5	Isoform 5 of Kallikrein-15	–	0.0423	–	Unknown
P05062	Fructose-bisphosphate aldolase B	–	0.0389	–	Energy metabolism
Q8NBI6	Xyloside xylosyltransferase 1	–	0.0259	–	O-glycan processing
P01833	Polymeric immunoglobulin receptor	–	0.0130	–	Neutrophil degranulation
O43933	Peroxisome biogenesis factor 1	0.0063	0.0118	–	Peroxisome orgnization
Q8N573-2	Isoform 2 of Oxidation resistance protein 1	–	0.0102	–	Stress response
P15924	Desmoplakin	–	0.0084	–	Neutrophil degranulation/Adherens junction organization
Down-regulated in naïve patients and up-regulated in treated patients					
C9JB55	Serotransferrin	1.4212	–	2.4371	Iron ion homeostasis/Platelet degranulation
P05452	Tetranectin	0.1457	–	0.1796	Ossification/Platelet degranulation
O75636-2	Isoform 2 of Ficolin-3	0.1364	–	0.2109	Complement activation
P49908	Selenoprotein P	0.0413	–	0.0380	Response to oxidative stress/Platelet degranulation
Q15848	Adiponectin	0.0390	–	0.0658	Glucose homeostasis

^a^Gene ontology is classified according to UniProt

^b^Not detected

### Proteins related to immune responses

**Fig. 4:**
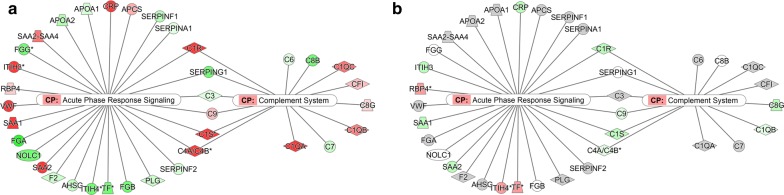
Proteins related to immune responses. Protein network analysis reveals proteins related to acute phase response signalling and the complement system in naive scrub typhus patients compared with normal subjects (**a**) and in patients treated with antibiotics compared with naive patients (**b**). Red indicates up-regulated proteins, and green indicates down-regulated proteins. The colour intensity corresponds to the degree of up- or down-regulation (fold change)

For more detailed investigation of proteins related to immune responses, protein network analysis was performed. The results clearly showed that expression levels of proteins involved in acute phase response signalling and complement system were up-regulated by infection with *O. tsutsugamushi* (Fig. 4a) and down-regulated by treatment with the antibiotic doxycycline (Fig. 4b). Acute phase response signalling is a rapid inflammatory response that provides protection against pathogens using non-specific defence mechanisms [26]. We also found that proteins involved in inflammation, such as serum amyloid proteins, protein S100-A8 and C-reactive protein, were up-regulated in naive patients (Table 3).

The complement system is part of the innate immune system that enhances the ability of antibodies and phagocytic cells to clear microbes and damaged cells from the host organism, promotes inflammation and attacks the plasma membrane of the pathogen [27]. Our results showed that, among the 32 differentially expressed proteins, six of those up-regulated were complement component proteins (Table 3). Proteomic analysis also revealed that the polymeric immunoglobulin receptor was exclusively expressed following infection with *O. tsutsugamushi* (Table 3). The polymeric immunoglobulin receptor facilitates the secretion of IgA and IgM [28]. It is well known that both IgM and IgG initiate complement system activation [29]. Therefore, infection by *O. tsutsugamushi* induces IgM production, and this may lead to the expression of complement component proteins and activation of complement system, resulting in activation of the innate immune system, including inflammation.

## Conclusions

In this study, we performed proteomic analysis of blood serum from scrub typhus patients to investigate the pathophysiological mechanisms and discover potential diagnostic biomarkers for scrub typhus infection. Comparative analysis revealed that proteins involved in immune responses and blood coagulation were dynamically regulated following infection with *O. tsutsugamushi* and subsequent antibiotic treatment. In particular, complement system was activated in scrub typhus patients. We also discovered proteins that are differentially expressed among normal subjects, naive patients and patients treated with antibiotics. To our knowledge, this is the first analytical study of clinical samples from scrub typhus patients. Our results provide valuable information for further investigation of scrub typhus therapy and diagnosis.

## Additional files

**Additional file 1: Figure 1.** Nested PCR of 56 kDa gene confirmed the infection of *O. tsutsugamushi* in scrub typhus patients. **Figure 2**. Functional annotation of the proteins identified in each group.

**Additional file 2: Table 1.** Total list of identified proteins.

## Abbreviations

ALT: alanine aminotransferase
aPTT: activated partial thromboplastin time
AST: aspartate aminotransferase
PBMC: peripheral blood mononuclear cell
FXR: frarnesoid X receptor
GeLC-MS/MS: one-dimensional sodium dodecyl sulfate–polyacrylamide gel electrophoresis followed by liquid chromatography-tandem mass spectrometry
INR: international normalized ratio
LXR: liver X receptor
PT: prothrombin time
RXR: retinoid X receptor
WBC: white blood cell
