# Supplementary material for: Clinical proteomic analysis of scrub typhus infection
Source: Clin Proteomics. 2018 Feb 14;15:6. doi: 10.1186/s12014-018-9181-5 (PMC5812041; doi:10.1186/s12014-018-9181-5)
Supplement: Supplementary file 1 — Additional file 1: Figure 1. Nested PCR of 56 kDa gene confirmed the infection of O. tsutsugamushi in scrub typhus patients. Figure 2. Functional annotation of the proteins identified in each group. [file 12014_2018_9181_MOESM1_ESM.pdf]

Additional file 1: Fig. S1

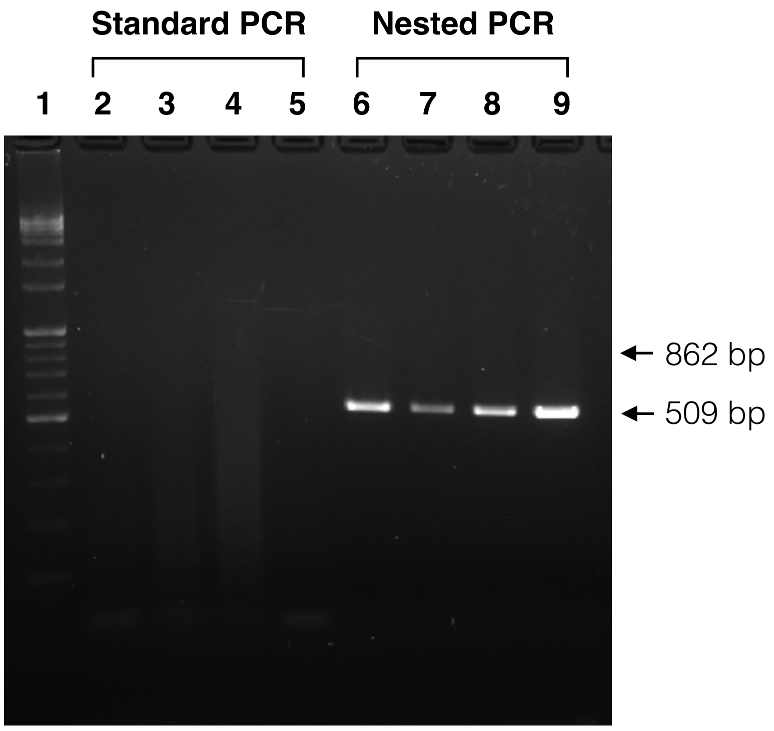

Lane 1: DNA size marker
Lane 2 & 6: Patient 1
Lane 3 & 7: Patient 2
Lane 4 & 8: Patient 3
Lane 5 & 9: Patient 4

Figure S1. Nested PCR of 56 kDa gene confirmed the infection of *O. tsutsugamushi* in scrub typhus patients. Clinical samples of patient 1, 2, and 4 were used for proteomic analysis.

Additional file 1: Fig. S2

TR/RXR activation
Ceramide biosynthesis

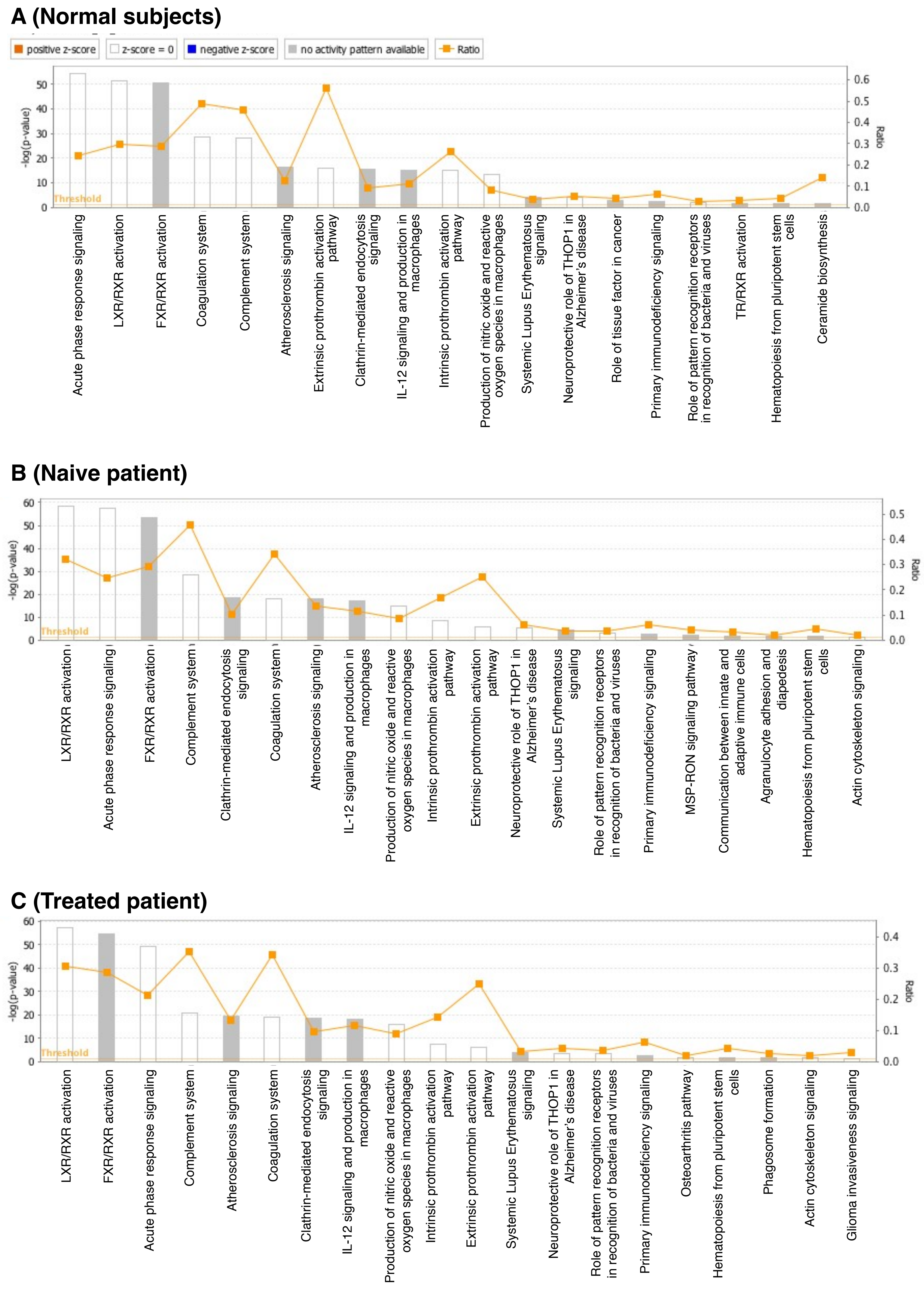

Figure S2. Functional annotation of the proteins identified in normal subjects (A), naive scrub typhus patients (B), and treated patients (C).
